# The development of the national tuberculosis research priority in Indonesia: A comprehensive mixed-method approach

**DOI:** 10.1371/journal.pone.0281591

**Published:** 2023-02-09

**Authors:** Trisasi Lestari, Ahmad Fuady, Finny Fitry Yani, I Wayan Gede Artawan Eka Putra, Ivan Surya Pradipta, Lidya Chaidir, Diah Handayani, Agus Fitriangga, Maria Regina Loprang, Imran Pambudi, Rovina Ruslami, Ari Probandari

**Affiliations:** 1 Center for Tropical Medicine, Faculty of Medicine, Public Health and Nursing, Universitas Gadjah Mada, Yogyakarta, Indonesia; 2 Department of Community Medicine, Faculty of Medicine, Universitas Indonesia, Jakarta, Indonesia; 3 Department of Public Health, Erasmus MC University Medical Center Rotterdam, Rotterdam, The Netherlands; 4 Primary Health Care Research and Innovation Center, Indonesian Medical Education and Research Institute, Faculty of Medicine Universitas Indonesia, Johar Baru, Jakarta, Indonesia; 5 Department of Child Health, Faculty of Medicine, Universitas Andalas, Padang, Indonesia; 6 Department of Public Health and Prevention Medicine, Faculty of Medicine, Universitas Udayana, Denpasar, Indonesia; 7 Department of Pharmacology and Clinical Pharmacy, Faculty of Pharmacy, Universitas Padjadjaran, Jawa Barat, Indonesia; 8 Department of Biomedical Sciences, Faculty of Medicine, Universitas Padjajaran, Jawa Barat, Indonesia; 9 Department of Pulmonology and Respiratory Medicine, Faculty of Medicine, Universitas Indonesia, Jakarta, Indonesia; 10 Department of Community Medicine, Faculty of Medicine, Universitas Tanjungpura, Kota Pontianak, Indonesia; 11 WHO Indonesia Office, Jakarta, Indonesia; 12 Ministry of Health Republic of Indonesia, Jakarta, Indonesia; 13 Department of Biomedical Science, Faculty of Medicine, Universitas Padjadjaran, Jawa Barat, Indonesia; 14 Department of Public Health, Faculty of Medicine, Universitas Sebelas Maret, Jawa Tengah, Indonesia; VART Consulting PVT LTD, INDIA

## Abstract

Ranked second in global tuberculosis (TB) incidence, Indonesia has developed a National Strategy for TB Prevention and Control 2020–2024 to accelerate the TB elimination program. Research and innovation are key pillars to support the program and need to be prioritised. This study aimed to develop updated national TB research priorities in Indonesia. This study was a mixed-methods study consisting of an open survey, a published literature survey, and Delphi survey. The open survey invited all related TB stakeholders to answer (a) the main barriers of the TB program and (b) the need for studies to support TB elimination. The published literature survey retrieved scientific articles published in national and international journals between 2015 and 2020 to identify gaps between published research and the current national strategy for TB control. The online survey and literature survey informed a panel of TB experts in a two-phase Delphi Survey to select the top 10 priority research topics. We identified 322 articles and analysed 1143 open survey responses. Through two-phases Delphi surveys, top ten research categories were listed: early TB detection; diagnosis and treatment of DR-TB; contact investigation; case detection and treatment of child TB; TB preventive therapy; government policy; laboratory for drug-sensitive- and drug-resistant-TB diagnosis; treatment adherence; diagnostic tool development; and community empowerment. This study also found the gap between stakeholders’ interests and the importance of translating research into policy and practice. TB research priorities have been identified through the involvement of various stakeholders. The combination of an online survey, a published literature survey, and a Delphi survey was a rigorous methodology and was fit to build a systematic consensus about the priority of TB research.

## Introduction

Research and innovation are critical pillars of the WHO’s End Tuberculosis (TB) Strategy [1]. Developing and improving the research and innovation environment are critical measures to achieve the three main targets in 2035, i.e., reducing TB incidence and mortality and eliminating TB-related catastrophic costs: the more TB research, the higher odds for better TB-related policies [2]. However, numbers are not the only important aspect. While TB high burden context has stimulated research interest with significantly increasing numbers of studies, achieving the national TB elimination targets urges the harmonisation of studies with a highly focused national agenda [3]. Therefore, TB research and innovation need to be prioritised and are integral to the research process. It is also important to allocate resources efficiently, stimulate debate, and strengthen stakeholders’ involvement and role in order to improve the TB research agenda.

Indonesia has the second-highest TB incidence worldwide and accounts for 8.5% of the global burden of TB despite the enormous efforts in the last two decades [4]. With COVID-19 disrupting health services in every part of the country, Indonesia is facing a significant drawback in TB case finding. In 2020, TB case finding dropped by 30% and only increased by 12% in 2021 [5]. This data reflects an increase in missing TB cases, which could result in additional TB deaths. Strategies and concerted efforts in identifying and treating these missing cases [6].

Indonesian researchers have produced TB-related research and scientific papers. The studies have supported the National Strategy for TB Prevention and Control, updated regularly every five years [7]. Despite the progress, some gaps—from what is targeted and produced—are inevitable. Diverse population characteristics, tuberculosis prevalence, and access to TB diagnosis and treatment facilities across the country also need to be considered in developing local and national policies for TB elimination [8]. Therefore, developing a national TB research priority is highly needed in eliminating TB in the country. Then, the priority should be bold, sharp, and accommodative to all aspects [9]. Finally, it should combine the needs of conducting research in basic sciences, clinical sciences, community, psychosocial, and health systems. It is essential to apply a robust method, involve as many stakeholders as possible, and guarantee transparency in the development process to develop such a bold research priority.

This study aimed to systematically develop the national TB research priorities in Indonesia with several objectives, i.e., (a) identifying gaps between current research achievement and the national targets, (b) collecting voices from all stakeholders on what studies should be prioritised, and (c) formulating the TB national research priorities.

## Methods and analysis

### Study design

The development of the Indonesian TB research priorities applied a mixed-method design to answer the three defined objectives. The study was a combination of deductive and inductive research approaches through three integrated parts: a published literature survey, online survey, and a two-stage Delphi survey. This study was conducted from December 2020 to August 2021.

The Indonesian Tuberculosis Research Network (JetSet TB Indonesia) initiated and led this study, supported by the National Tuberculosis Program (NTP) at the Ministry of Health (MoH) and the Indonesian Tuberculosis Expert Committee. JetSet TB Indonesia shared the activities of this project among its committee members for published literature survey (led by ISP), online surveys (AF), and Delphi surveys (TL) activities. Consultation with partners from the MoH, TB expert committee, and the WHO country office of Indonesia was conducted before proceeding to the next stage of the research priority-setting process. The NTP approved the final report prior to its publication to a broader TB community that could benefit from this work.

### Published literature survey

A survey of published literatures on TB from studies in Indonesia identified research gaps between TB-related studies published in scientific journals and what is targeted in the National Strategy for TB Prevention and Control 2020–2045 [7]. We systematically searched original articles, systematic reviews, or meta-analyses related to TB that had an Indonesian context. The articles indexed in PubMed and Embase databases and published between 2015 and 2020 were collected. The search terms can be seen in the Text in S1 Appendix. Narrative reviews, book chapters, clinical case reports, and case series publications were excluded. In addition, we did hand-searching to collect scientific articles published in the national database. To cover the excellent quality of the national publications, we conducted hand searching in the top national journals accredited in SINTA 1, the highest level of Indonesian journal accreditation (https://sinta.ristekbrin.go.id).

After removing duplicated articles, the list of articles was screened based on the title, abstract, and full text, if needed, by seven reviewers (TL, AF, ISP, FFY, IWGAEP, LC, and DH). The reviewers grouped the articles based on themes described in the National Strategy for TB Prevention and Control to identify the gaps (See Table 1) [9]. Discrepancies between reviewers’ judgments were resolved in a group meeting.

**Table 1:** Themes and pre-determined categories based on the national TB control strategy.

Themes	Pre-defined categories
Strategy 1: Strengthening commitment and leadership to accelerate TB elimination by 2030	Governance
	Leadership
Strategy 2: Improving access to high-quality and patient-centred TB services	Access to TB care
	Quality of TB care
	Patient-centeredness
Strategy 3: Optimising health promotion and TB prevention, TB preventive therapy, and infection control	Health promotion
	TB prevention
	TB infection control
Strategy 4: Translating research and technology for screening, diagnosis, and treatment	TB screening
	TB diagnosis
	TB treatment
Strategy 5: Increasing community and partner engagement in TB elimination	Community partnership
	Public-private mix
Strategy 6: Improving TB program management through health system strengthening	Health system strengthening
	TB financing system

### Online survey

We deployed an online-based survey from 22 November to 7 December 2021. The survey was open to all TB-related stakeholders who have had at least one year of experience related to TB program, research, care, or project. The stakeholders represent components of potential actors as identified in the high-quality health system framework [10]. In collaboration with NTP and TB coordinators in 34 provinces of Indonesia, we applied a purposive and snowballing sampling method and distributed the invitation to prospective respondents at the national, province, district, and facility levels. We reached TB researchers through the JetSet TB Indonesia network and applied a snowballing technique to other TB researchers in their network. We also invited drug-sensitive (DS) and drug-resistant (DR) TB patients who were treated in the continuation phase or completed the treatment and their families through TB patients’ community organisations (*Perhimpunan Organisasi Pasien TB Indonesia*, POP TB).

The questionnaire was developed in collaboration with the NTP, the TB expert committee, and the WHO country office of Indonesia. For healthcare workers, researchers, and NTP officers, the questionnaire consisted of two main questions. First, in a multiple-choice question, they were asked to choose one type of study or research topic that they think the most potential to accelerate TB elimination in Indonesia (epidemiology, diagnosis, treatment, prevention, program management, health (financing) system, basic science, vaccine and drug development, and another research topic). Second, in an open-ended question, they were asked to describe (a) the main barriers to implementing TB programs or research and (b) the most critical strategy to solve the problem and improve the TB program or research to accelerate TB elimination in Indonesia.

For people with TB and their family members, we developed the different structures and questions since they may not be familiar with the research term. First, in a multiple choice question, we asked them the choice of a problem they have ever faced when accessing TB-related services. Second, in an open-ended question, we ask them to describe their suggestions to resolve the problem the faced.

The survey questionnaire and data were collected and managed using REDCap, electronic data capture tools, hosted at the Faculty of Medicine Universitas Indonesia [11]. The questionnaire was tested on 20 individuals who fit the inclusion criteria for the prospective respondent (see Table in S2 Appendix).

We used the National TB Elimination Strategy 2020–2025 to guide the analysis of survey results and ensure that the research priorities aligned with the national strategy for TB elimination. The framework consists of six main dimensions, which are:

1. the political commitment of the national and local government,
2. access to quality and patient-focus TB services,
3. optimized TB awareness, prevention, and infection control,
4. use of research and technology for screening, diagnosis, and management of TB,
5. improvement in the role of TB stakeholders, and
6. improvement in TB management through health system strengthening initiatives.

Duplicate entries were identified by comparing the respondent’s name and email address. In case of duplicate entries, an entry with the most completed data will be included for analysis. Respondents who only completed the personal information part and did not respond to any of the key questions were excluded from the analysis. Open and axial coding was conducted by two researchers using NVivo Version 12 (QSR International Pty Ltd.) software. Thematic analysis was conducted to identify key messages that emerged repeatedly within the data and grouped them into sub-categories, categories, and themes and derive meaning from them.

### Delphi survey

We organized a two-phase Delphi survey and purposively selected health experts into a panel. The experts were selected based on their experience and expertise in public health, clinical medicine, and TB control programs, including:

1. Senior TB program manager at the national, province, or district level
2. Medical specialist in internal medicine, paediatric, radiology, or pulmonology
3. Registered nurse, midwife, or pharmacist who has experience in TB care at least one year
4. Researcher or lecturer in the field of medicine, health or social sciences related to TB who holds a master or Philosophy Doctor degree
5. Senior TB project manager at national Non-Governmental Organisation

The NTP invited prospective experts to attend a virtual meeting, informed them of the open survey result, and asked them to select research topics priorities in a two-round of Delphi survey. We provided an online questionnaire (using Qualtrics XM, Provo, UT platform) for them to select the priorities in the panel meeting. Participation in the Delphi survey was anonymous, voluntary, and unpaid. The experts gave their written consent before proceeding with the survey, and they could withdraw from the survey. The Delphi group size does not depend on statistical power but on group dynamics to reach a consensus among experts.(12) We invited 60 experts to the survey to ensure that 10–20 experts were available in the second phase to draw a consensus [12, 13].

#### Delphi survey phase 1

The Delphi survey phase 1, conducted on 4–5 August 2021, was to select the top ten research topics priority out of 29 proposed research topics. Based on the published literature survey and online survey results, we identified 29 research topics. Out of 29 research topics, 19 have sub-topics. Experts were asked to select 10 out of 29 research topics. For each selected research topic that contains subtopics, we asked the respondent to select one sub-topic priority. Each selected research topic and sub-topic were scored 1. We summed the scores for each topic and sub-topic and listed the top ten topics. The new list was established for the Delphi survey phase 2. All experts had the opportunity to give written comments on the process or proposed research topic, which was not included.

#### Delphi survey phase 2

**Fig 1:**
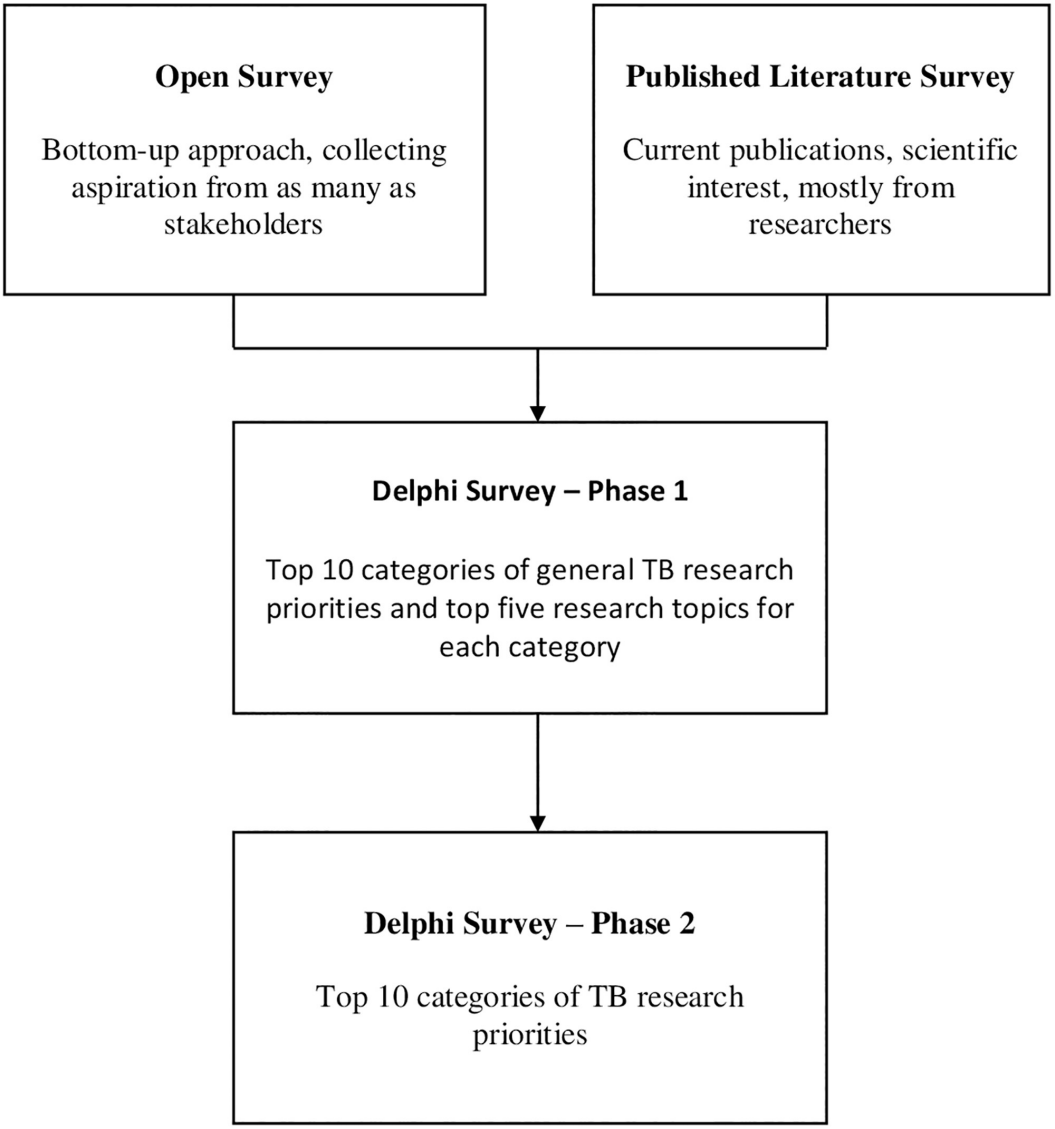
Ethics and dissemination.

The Delphi survey phase 2, held on 9 August 2021, was to rank the selected research topics. After the Phase 1 survey, we identified 15 research topics with the highest scores instead of ten research topics as initially planned. The 15 topics were presented to the same experts. We asked them to rank each topic from 1 (the highest) to 15 (the lowest) by considering the topics’ feasibility, importance, and available evidence. Topics were scored with the opposite value of the ranking value. A topic that ranked 1^st^ was scored 15, and a topic that ranked 15^th^ was scored 1. The total score for each topic was summed up and sorted from the highest to the lowest. The top ten topics with the highest score were selected as the research priority topics (Fig 1).

In the open survey, a description of the study and ethical considerations was provided on the first page of the online questionnaire before participant giving their written consent to participate in the survey. In the Delphi Survey, although invited experts met during an information session in a virtual room, their responses were confidential and anonymised to secure their independence and avoid biases by identities or pressures of others, including the investigator. The Ethical Committee at the Faculty of Medicine Universitas Sebelas Maret Surakarta, Indonesia, granted ethical clearance (No. 168/UN27.06.6.1/KEPK/EC/2020).

## Results

### Published literature survey

Of 814 articles retrieved from databases, 322 articles met the criteria for review and analysis. The articles were grouped into six dimensions, which were TB-related services (213 articles, 66%), policy and regulation (55, 17%), multisector and intersectional programs (101, 31%), human rights (21, 6%), health financing (82, 25%), and others (187, 58%). There was no study corresponding directly to the Indonesian National TB Strategy. Full review processes are documented (See: https://osf.io/h2qyf/?view_only=26a898a8153b4fa18c311f1e03e25b14).

### Online survey

Out of 1918 responses submitted, we removed 775 (40.4%) responses: 553 (28.8%) respondents consented to fill the questionnaire but didn’t answer any single question, 109 (5.7%) did not answer questions on research priority, and 113 (5.9%) responses were not valid or duplicates. A total of 1143 (59.6%) responses were analysed, with the majority of response coming from Java (52.8%), the most populous island in Indonesia, followed by Bali (14.6%) and other islands (32.5%) (Table 2). Most respondents were TB-related health care workers (37.9%) and NTP program officers (33.9%) in 31 out of 34 provinces in Indonesia.

**Table 2:** Characteristics of online survey respondents.

Characteristics	n	%
*Group*		
Individual patient	53	4.6
Family members of person with TB	17	1.5
TB-related NGOs and international organisation	63	5.5
NTP Program officers	388	33.9
National health insurance agency (BPJS)	3	0.3
Local government	31	2.7
Public and private hospital managers	7	0.6
Primary Health Care (PHC) managers	36	3.1
Researchers in universities and research institutions	58	5.1
TB-related healthcare workers	433	37.9
TB logistics, pharmacy, and distribution network	10	0.9
Others	44	3.8
*Region of Indonesia*		
Western	804	70.3
Central	283	24.8
Eastern	55	4.8
*Islands*		
Java	604	52.8
Bali	167	14.6
Other	371	32.5

There were no dominant research topics suggested among researchers. Respondents among non-researchers and people with TB and their families suggested more studies in TB prevention, respectively, 35.8% and 38.6%, followed by studies in program and healthcare management (21.3%) and TB-related services (15.7%) (Table 3). From the survey results, we created 29 categories and 65 sub-categories of TB research topics. The summary of the categories and sub-categories of the top 10 TB research topics is available in Table in S3 Appendix).

**Table 3:** Research topics selected by respondents.

Research topic selected	Researchers		Non-researchers		Person with TB and family members	
	n	%	n	%	n	%
Epidemiology	11	19	112	11	N/A	N/A
Diagnosis	8	14	67	7	9	13
Treatment	6	10	118	12	7	10
Prevention	10	17	363	36	27	39
Program and healthcare management	11	19	216	21	11	16
Healthcare and health financing system or TB-related costs	3	5	65	6	2	3
Basic science	0	0	7	1	N/A	N/A
Vaccine and drug development	6	10	31	3	N/A	N/A
TB-related stigma	N/A	N/A	N/A	N/A	6	9
Other topics	3	5	25	3	2	3
Did not know	0	0	10	1	6	9

**N/A**, Not applicable or not asked to the respondent population.

### Delphi survey

Of 60 experts invited to the Delphi Survey, 28 (47%) and 25 (42%) experts joined Phase 1 and 2, respectively. The experts represented researchers from universities (11 in Phase 1; 8 in Phase 2), NGOs (8; 5), TB expert committee (5; 3), Ministry of Health/NTP (4; 5), medical association (1; 3), and penitentiary—representing specific community (1; 1).

Table 4 summarises the top ten priorities after the overall process, from the published literature survey and open survey towards consensus in Delphi survey Phase 2. The highest score for top research priority was early TB detection, followed by diagnosis and treatment of drug-resistance (DR)-TB. The high scores for these two topics were in accordance with the suggestion from the open survey (413 and 263 responses, respectively) and interest in publication (10 and 17 published articles). The third, fourth, and fifth priorities were contact tracing, case detection and treatment of child TB, and TB preventive therapy, with few publications.

**Table 4:** Top 10 Indonesian national TB research priorities.

Domains	Open survey	Literature survey	Delphi Phase 1	Score in Delphi Phase 2	Priority
**Strategy 1: Strengthening commitment and leadership to accelerate TB elimination by 2030**					
Government policy	49	1	13	116	(6)
**Strategy 2: Improving access to high-quality and patient-centred TB services**					
Early TB detection	413	10	22	241	(1)
Access to TB services	63	0	5		
Laboratory for DS- and DR-TB diagnosis	75	0	13	85	(7)
TB treatment adherence	156	16	13	74	(8)
Diagnosis and treatment of DR-TB	263	17	18	182	(2)
Diagnosis and treatment of TB with comorbidities	19	23	7		
Case detection and treatment of child TB	27	9	16	139	(4)
Risk factors of DS- and DR-TB	17	21	8		
**Strategy 3: Optimising health promotion and TB prevention, TB preventive therapy, and infection control**					
TB infection control in health facilities	104	0	1		
Environment and lifestyle	74	8	6		
TB preventive therapy	119	7	16	127	(5)
Contact investigation	103	5	17	174	(3)
**Strategy 4: Translating research and technology for screening, diagnosis, and treatment**					
Digital technology for TB management	69	4	10	25	
Evaluation and development of policy and coordination between ministries and research institutions	19	0	3		
Development of basic science, genetics, clinical, and implementation research	4	108	6		
TB vaccine development	37	2	8		
TB drug development	69	35	8		
Diagnostic tools development	16	9	12	47	(9)
**Strategy 5: Increasing community and partner engagement in TB elimination**					
Community empowerment	504	3	12	45	(10)
Coordination with related stakeholders	50	3	11	32	
Feedback from community	59	2	9	34	
Reducing stigma and discrimination	17	1	7		
**Strategy 6: Improving TB program management through health system strengthening**					
Commitment and intersectoral coordination	35	0	8		
Improving human resources and competence	89	4	3		
Integrated TB recording and reporting	26	0	11	42	
Health financing for TB program	69	11	5		
TB surveillance	82	0	10	9	
TB logistic management	31	0	2		

Grey highlighted indicates the high number of respondents’ suggestions in an open survey, articles published, and priorities given in the Delphi survey.

Development of basic science, genetics, clinical, and implementation research was not included in the top ten priorities despite its very high number of publications. Only four respondents and six experts suggested this topic as a priority. TB drug development was also not prioritised despite the high number of publications.

## Discussions

This study highlighted the importance of developing national TB research priorities through a bottom-up approach involving as many TB-related stakeholders as possible. This approach helped (a) identify gaps in published studies and the national targets’ needs and (b) collect stakeholders’ and experts’ opinions for research needs—all those results combined to formulate the TB national research priorities. Both stakeholders’ and experts’ opinions consistently constructed the top two research priorities formulated by results from open survey and publication interests. The other priorities indicated the remaining gaps: high aspirations from open literature surveys and Delphi surveys but limited publications.

The scope of research on TB is vast. However, resources for research are limited, and there is an urgent need to strengthen the national TB control to accelerate the progress of the National TB program after a low performance during the COVID-19 pandemic in 2020 and 2021. In addition, supporting TB research has been identified as one of the global priority strategies to end TB.

To our knowledge, this is the first comprehensive study combining inductive and deductive approaches to develop the Indonesian national TB research priorities agenda. This study explored ideas from healthcare practitioners, researchers, implementers of TB control programs from 31 provinces of Indonesia, and patients and their family members to represent the service users’ voices according to their experiences accessing TB-related services. This approach completed the findings from the literature before inviting experts’ opinions. This current methodology was rigorous yet transparent while maintaining the anonymity of all respondents. Identifying research priorities from those working in the field with the necessary knowledge, experience, and expertise as to what research priorities for TB control programs should be of utmost importance [14–16].

The top-five TB research priorities indicate the importance of a comprehensive approach: early detection, contact investigation, diagnosis, and TB treatment. Increasing TB case detection and contact investigation is inevitable, given Indonesia’s remaining high TB incidence. Only a tiny proportion of people develop TB after contracting *Mycobacterium tuberculosis*. About 50% of those who contracted the bacteria are latently infected but remain well, with the potential for developing TB disease during their lifetime [17–20]. Therefore, TB (active) case finding, early diagnosis, contact investigation, and TB preventive treatment are critical measures in the TB elimination program. The proposed research priorities are in line with the Global Plan to End TB 2018–2022 to (1) reach at least 90% of all people who need TB treatment and prevention, (2) reach at least 90% of people in key populations, and (3) achieve at least 90% treatment success among people diagnosed with TB or who are eligible for preventive therapy [21].

Drug-resistant TB (DR-TB) was a critical population that should be addressed sufficiently, given that Indonesia is among ten countries contributing to about 70% of the estimated global incidence of MDR/RR-TB. While DR-TB treatment is costly, the vital strategy is to prevent its development by improving the quality of drug-sensitive TB (DS-TB) services in primary healthcare services and increasing access to drug susceptibility tests [22–25]. These concerns were indicated by suggestions provided by respondents and experts in this study.

This study also identified the high number of publications but low interest indicated by voices in the survey. There are a significantly higher number of publications in basic science, genetics, clinical, and implementation research than the other topics, but very few suggestions to prioritise the topics. There are several plausible reasons. First, basic science, clinical, and implementation research have been conducted by universities with adequate funding and have a high likelihood of scientific publication. However, second, the impact of the basic research on TB practical management is limited or needs a much longer time for the outcome [26–28]. Third, NTP program officers, who were the majority of the open survey respondents, were not familiar with the research topics. These indicate a problem in translating research into policy and practice. It should also be resolved to maximise the impact of research on the community.

The online survey in this study was the first nationwide survey that involved all stakeholders from 31 provinces of Indonesia. Although more than half of the respondents were those living on Java Island, the survey still provides critical input since 60% of TB cases in Indonesia have been reported from provinces on Java. This survey accommodated voices that were not heard from the previous approach. The development of previous TB research priority had been made explicitly for implementation research and only involved the experts in the process [29]. This study allowed all stakeholders to share their ideas for future TB research. In addition, a published literature survey of current published evidence on TB research in Indonesia has helped provide a master list of priorities. Finally, it informed the expert panel about gaps between evidence and practice.

The Delphi survey is a relevant source of evidence in healthcare research. The survey has been used to identify research priorities in many countries and health subjects [30–32] and allows access to a wide range of experts without spending considerable resources. Furthermore, this method also allows iteration around the topics in question. It is also an essential method for developing consensual guidance on best practice and exploration of a field beyond existing knowledge and the current conceptual framework [33]. A limited number of experts participating in the Delphi survey may not adequately represent the full spectrum of TB stakeholders in Indonesia. However, a low response rate is common in Delphi surveys. Experts may also have particular research interests that could have affected their prioritisation of topics. Sharing the online survey results and literature survey with the expert panel prior to the Delphi survey should reduce this potential bias.

The worldwide incidence of coronavirus disease 2019 (COVID-19) has been significantly affecting the achievement of the National TB program and the provision of TB care to patients in 2020 and 2021. Research activities across Indonesia were affected, with universities and government offices closing, travel restrictions, and researchers working from home where possible. Progress of the pandemic until the first quarter of 2022 indicates that COVID-19 disease will stay for long and will continue affect how and what TB research will be conducted to accelerate the performance of TB program in the country [34]. Listening to voices from a wide range of stakeholders will help policymakers better identify barriers and potential interventions that implementers need in the field. The research community should also adapt and create a better way to do research that would benefit TB patients and the community.

## Conclusions

The approach combining an online survey, published literature survey, and Delphi survey helped identify gaps between published studies and the need to achieve national TB targets and collect stakeholders’ and experts’ opinions for research needs. The current research achievement has not addressed every component of the national TB strategies. The first two highest scores of the top research priority, i.e., early TB detection/diagnosis and treatment of (DR)-TB, are in synergy with the stakeholders’ voices. The topic of contact tracing, case detection and treatment of child TB, and TB preventive therapy is perceived as priorities by the stakeholders; however, the number of publications is lacking. On the other hand, some other topics of TB research with a high number of publications (e.g., basic and clinical research) are not perceived as a priority for the national TB research agenda.

The findings have generated the top research priorities to accelerate the elimination of TB in Indonesia. This approach also allowed us to find the gap between stakeholders’ interests and the importance of translating research into policy and practice. Sharing these results among key stakeholders at the national and international levels could inspire TB researchers to address some of these research priorities. Since implementers of TB programs and policymakers’ perspectives may dominate the view in developing the priorities, we suggest that further study identify potential research priorities from the perspective of clinicians and patients.

## Supporting information

S1 Appendix Search terms of published literature survey. (DOCX)

S2 Appendix Categories and sub-categories of TB research topics in the online Delphi survey and published literature survey. (DOCX)

S3 Appendix Categories and sub-categories of research topics from Delphi survey, open survey and published article survey. (DOCX)
