# Supplementary material for: The development of the national tuberculosis research priority in Indonesia: A comprehensive mixed-method approach
Source: PLoS One. 2023 Feb 9;18(2):e0281591. doi: 10.1371/journal.pone.0281591 (PMC9910756; doi:10.1371/journal.pone.0281591)
Supplement: S1 Appendix — (DOCX) [file pone.0281591.s001.docx]

**S1 Appendix. Search terms of published literature survey**

1. PubMed

((tuberculosis[MeSH Terms]) OR (tuberculosis[Title/Abstract]) AND ((indonesia[MeSH Terms]) OR (indonesia[Title/Abstract])

1. Embase

(('tuberculosis'/exp OR tuberculosis:ab,ti) AND ('indonesia'/exp OR indonesia:ab,ti)) AND 'article'/it

1. Hand searching in the national medical journals

“tuberkulosis”; “tuberculosis”; “tb”
