# Supplementary material for: The development of the national tuberculosis research priority in Indonesia: A comprehensive mixed-method approach
Source: PLoS One. 2023 Feb 9;18(2):e0281591. doi: 10.1371/journal.pone.0281591 (PMC9910756; doi:10.1371/journal.pone.0281591)
Supplement: S2 Appendix — (DOCX) [file pone.0281591.s002.docx]

**S2 Appendix**. **Categories of respondents based on the high-quality health system framework.**

| **Components** | **Potential Actors** | **Target population** |
| --- | --- | --- |
| Populations | Individual patient | DS- and DR-TB patients |
|  | Patient’s family member | DS- and DR-TB patients’ family member/parent |
|  | Community or NGOs | Religion-based NGOs (e.g., Aisyiah, LKNU), Patient-based NGOs (e.g., POP TB), Community-based NGO (e.g., PPTI) |
| Governance | The NTP | All NTP staff and related function/department |
|  | Provincial TB Program | TB Coordinator, head of CDC department |
|  | District TB Program | TB Coordinator, head of CDC department |
|  | Ministry of Research and Technology | TB and infectious disease researchers |
|  | National health insurance agency (BPJS) | BPJS staff (national and province-level) |
|  | Local government | Regional Development Planning Board (Bappeda)  Head of district health office  Head of provincial health office |
| Platform | Public hospital | Hospital manager, TB program officers |
|  | Private hospital | Hospital manager, TB program officers |
|  | Primary Health Care (PHC) | Head of PHC, TB program officers |
|  | University | TB Researchers  University Research and Community Development Unit (LP2M) |
|  | Research institutions | Researchers at NIHRD, Eijkman Institute, The Indonesian Institute of Science (LIPI), Regional Research and Development Agency |
|  | TB-related international organisations | WHO, KNCV, Global Fund |
| Workforce | Clinicians | TB doctors at PHC and hospital, specialists (internist, paediatrician, pulmonologist, radiologists) |
|  | Nurses | TB nurses at PHC and hospital |
|  | Midwives | Community midwives |
|  | Laboratories | Microbiologist, Laboratory technician at PHC, hospital, and regional health laboratory centre |
|  | Pharmacists | Pharmacist and pharmacy-assistant |
|  | Professional organisation | PAPDI, PDPI, IDAI, IDUI, Hifarsi, PPNI, IBI |
| Tools | TB logistics and distribution network | Regional pharmacy unit (IFK), other logistics channels |
|  | TB information system | SITB/SITT manager, TB data statisticians, TB data officers at district and province level |
