# Supplementary material for: The development of the national tuberculosis research priority in Indonesia: A comprehensive mixed-method approach
Source: PLoS One. 2023 Feb 9;18(2):e0281591. doi: 10.1371/journal.pone.0281591 (PMC9910756; doi:10.1371/journal.pone.0281591)
Supplement: S3 Appendix — (DOCX) [file pone.0281591.s003.docx]

**S3 Appendix. Categories and sub-categories of research topics from Delphi survey, open survey and published article survey.**

| **No** | **Category** | **No** | **Sub-category** | **Number of supporting response from** | | |
| --- | --- | --- | --- | --- | --- | --- |
|  |  |  |  | **Delphi survey** | **Open survey** | **Published articles** |
| 1 | Optimizing TB active case finding | 1 | Active case finding in high risk population | 8 | 209 | 3 |
|  |  | 2 | Development of method for screening and active case finding in high risk population | 6 | 43 | 3 |
|  |  | 3 | Development and implementation of algorithm for TB diagnosis | 2 | 69 | 3 |
|  |  | 4 | Development or evaluation of integrated TB diagnosis and treatment services with other healthcare services | 1 | 85 | 0 |
|  |  | 5 | Development of digital technology for active case finding | 2 | 7 | 1 |
| 2 | Optimizing TB drug-resistant diagnosis and treatment | 6 | Evaluation or improvement of TB treatment compliance and adverse events management | 6 | 143 | 5 |
|  |  | 7 | Evaluation of enabler or support, including psychosocial support, for people with TB | 5 | 66 | 1 |
|  |  | 8 | Evaluation and improvement of comprehensive TB drug-resistant service | 3 | 19 | 5 |
|  |  | 9 | TB services network, including with private healthcare services | 1 | 2 | 5 |
|  |  | 10 | Evaluation and improvement of health care workers' capacity in providing TB drug-resistant management | 1 | 33 | 1 |
| 3 | Evaluation and intensification of contact investigation | 11 | Innovation and Strategy for an effective contact tracing | 9 | 18 | 1 |
|  |  | 12 | Evaluation and intensification of contact investigation | 7 | 85 | 4 |
| 4 | Strengthening pediatric TB case detection and treatment | 13 | Evaluation and improvement of TB diagnostic and management capacity | 15 | 23 | 9 |
|  |  | 14 | Provision and utilization of children-friendly TB drug-resistant treatment | 0 | 4 | 0 |
| 5 | Improvement of TB preventive therapy | 15 | Improvement of TB preventive therapy management | 3 | 57 | 2 |
|  |  | 16 | Improvement of materials for education and TB preventive therapy | 3 | 37 | 0 |
|  |  | 17 | Epidemiology of latent TB in Indonesia | 3 | 21 | 2 |
|  |  | 18 | Improvement of latent TB diagnostic capacity | 2 | 3 | 3 |
|  |  | 19 | Effectiveness of TB preventive therapy for people with latent TB infection | 3 | 1 | 0 |
| 6 | Central and local policy for National TB Control Strategy | 20 | Financing for TB services by local government and national insurance agency | 4 | 33 | 0 |
|  |  | 21 | Strengthening province- and district -levels' capacity to implement the the TB local action plan | 4 | 10 | 0 |
|  |  | 22 | Advocation and regular coordination meeting between local government and other stakeholders for harmonizing health-related regulations | 3 | 5 | 1 |
|  |  | 23 | Advocation to Internal Affairs Ministry and other ministries to monitor and evaluate the TB program | 0 | 1 | 0 |
| 7 | Optimizing laboratories for sensitive- and resitant TB drug diagnosis | 24 | Improvement of access to TB-related laboratories | 1 | 28 | 0 |
|  |  | 25 | Strengthening lab management, data quality, quality insurance, infrastructure, and monitoring, evaluation, and evaluation of TB diagnosis | 9 | 47 | 0 |
| 8 | Improving TB treatment compliance | 26 | Development of methods and innovation to improve TB treatment compliance | 6 | 117 | 1 |
|  |  | 27 | Incentives, rewards, or enablers for healthcare workers and TB drug observers | 2 | 33 | 3 |
|  |  | 28 | Lost-to-follow-up TB patients | 5 | 6 | 12 |
| 9 | Development of TB diagnostic tools | 29 |  |  |  |  |
| 10 | Community empowerment through communication, informatio, and education materials | 30 | Evaluation of Communication, information and education | 8 | 332 | 3 |
|  |  | 31 | TB Evaluation and increasing people engagement in TB prevention and control | 4 | 172 | 2 |
| 11 | Improving the quality of integrated and continuous TB recording and report system | 32 |  |  |  |  |
| 12 | Evaluating and improving communities' feedback | 33 | Evaluation of TB service quality in healthcare facilities | 5 | 50 | 2 |
|  |  | 34 | Evaluation of access to TB services | 4 | 9 |  |
| 13 | Evaluating and improving the coordination between TB program and relevant stakeholders | 35 | Role of pharmacies, filantrophy institution, zakat institutions, and corporate social responsibility agencies to support TB program | 4 | 22 | 3 |
|  |  | 36 | Evaluation of TB program coordination and management, from central to local level | 6 | 28 |  |
| 14 | Utilizing of digital technology in TB program | 37 | Digitalization and utilization of TB recording and reporting system | 6 | 38 | 3 |
|  |  | 38 | Simplification of TB recording and reporting system | 2 | 20 |  |
|  |  | 39 | Development of integrated TB recording and reporting system with other disease reporting systems | 1 | 2 | 1 |
|  |  | 40 | Development of digital technology to improve TB treatent compliance | 1 | 9 | 3 |
| 15 | Improving TB surveillance | 41 |  |  |  |  |
| 16 | TB drug development | 42 |  |  |  |  |
| 17 | Evaluating the commitment and cooperation between programa and ministries | 43 |  |  |  |  |
| 18 | Improving TB diagnosis and treatment coverage with comorbidities | 44 | Integration and coordination of TB-HIV services | 0 | 7 | 5 |
|  |  | 45 | Evaluation and improvement of integrated TB services with other comorbidities | 5 | 12 | 18 |
| 19 | Exploring risk factors of TB drug-sensitive and resistant | 46 |  |  |  |  |
| 20 | TB vaccine development | 47 | Development of new TB vaccine for children and adults for a better protection against TB | 7 | 26 | 1 |
|  |  | 48 | Evaluation of TB vaccine management, including its ditribution and utilization | 1 | 11 | 1 |
| 21 | Developing basic, genetic, and clinical, and implementation research for TB in Indonesia | 49 |  |  |  |  |
| 22 | Reducing TB-related stigma and discrimination | 50 |  |  |  |  |
| 23 | Environment and life style factors of TB development | 51 | Environment management to prevent TB infection | 0 | 23 | 8 |
|  |  | 52 | Effects of individual and life-style to TB infection. | 5 | 51 | 20 |
| 24 | Strengthening financing system for TB control program | 53 | Evaluation and advocation to central and local government for financing TB program | 2 | 22 |  |
|  |  | 54 | Evaluation and strengthening TB financing through national insurance program | 2 | 9 | 2 |
|  |  | 55 | Evaluation and mapping of potential financing for TB program | 0 | 8 | 1 |
|  |  | 56 | Evaluation of financing TB-related health and social problems | 1 | 18 | 1 |
|  |  | 57 | Evaluation of financing community activities and community health workers to support TB control program | 0 | 12 | 7 |
| 25 | Improving access to TB-related services | 58 |  |  |  |  |
| 26 | Evaluating and improving the policy and coordination between Ministry of Health and TB-related research institution | 59 | Utilization of TB program data for research and policy making | 3 | 18 |  |
|  |  | 60 | Development and mapping of TB research network in Indonesia | 0 | 1 |  |
| 27 | Evaluating and improving TB-related human resources | 61 | Evaluation of the availability, workload, and needs of TB-related human resources | 2 | 29 |  |
|  |  | 62 | Evaluation and improvement of TB-related human resources' capacity | 0 | 31 | 1 |
|  |  | 63 | Policy analysis related to healthcare staffs' rotation, mutation, and remuneration | 1 | 29 | 3 |
| 28 | Evaluating and strengthening TB logistic management | 64 | Evaluation and coordination between Health Offices, healthcare facilities, and pharmacies in all level. | 0 | 13 |  |
|  |  | 65 | Evaluation of TB program logistics planning, mapping, and management | 2 | 18 |  |
| 29 | TB infection control in healthcare facilities | 66 |  |  |  |  |

**Research topics proposed by stakeholders related to Strategy 1. Strengthening commitment and leadership to accelerate TB elimination by 2030**

|  | **Category** |  | **Sub-categories** | **Number of online survey participants support** | **Number of previous relevant research from published literature survey** |
| --- | --- | --- | --- | --- | --- |
| 1 | Research on policy in national, provincial and district level to implement national TB control strategy | 1 | Strengthening capacity of provincial and district in the implementation of action plan | 10 |  |
|  |  | 2 | Financing of TB treatment and other TB program activities by local government and Social Security (BPJS) | 33 |  |
|  |  | 3 | Regular advocacy and coordination between provincial/district government and stakeholders to harmonize health regulation | 5 | 1 |
|  |  | 4 | Advocacy to Ministry of Domestic Affairs and other Ministries to monitor and evaluate TB program | 1 |  |

1

**Research topics proposed by stakeholders related to Strategy 2. Improving access to high-quality and patient-centred TB services**

|  | **Category** |  | **Sub-categories** | **Number of online survey participants support** | **Number of previous relevant research** |
| --- | --- | --- | --- | --- | --- |
| 1 | Research to optimize TB case finding | 1 | Research to optimize active case finding among high-risk population | 209 | 3 |
|  |  | 2 | Development of TB screening methods | 43 | 3 |
|  |  | 3 | Development of TB diagnosis algorithm | 69 | 3 |
|  |  | 4 | Development/evaluation of integrated TB diagnosis and treatment services with other health services | 85 | 0 |
|  |  | 5 | Development of digital technology related to TB active case finding | 7 | 1 |
| 2 | Research to improve access to TB services | 1 | Innovations to improve TB access | 63 | 0 |
| 3 | Research to optimize laboratory for drug-sensitive and resistant TB | 1 | Research to improve access to TB laboratory | 28 | 0 |
|  |  | 2 | Research to strengthen lab management | 47 | 0 |
| 4 | Research to improve TB treatment adherence | 1 | Research on incentives, reward, enablers for improving treatment adherence | 33 | 3 |

2

|  |  | 2 | Research and innovation related to methods of treatment observation to improve treatment adherence | 117 | 1 |
| --- | --- | --- | --- | --- | --- |
|  |  | 3 | Research related to lost to follow up TB patients | 6 | 12 |
| 5 | Research to optimize diagnosis and treatment among drug resistant TB patients | 1 | Research and evaluation on comprehensive drug resistant TB service | 19 | 5 |
|  |  | 2 | Research to evaluate enabler or other supports to drug resistant TB treatment | 66 | 1 |
|  |  | 3 | Research on TB service network including the network with private healthcare, and primary health care facilities | 2 | 5 |
|  |  | 4 | Research to evaluate or improve treatment adherence and side-effect management | 143 | 5 |
|  |  | 5 | Research on evaluation and development of capacity of health staff on drug resistant TB management | 33 | 1 |
| 6 | Research to improve coverage of diagnosis and treatment of TB with comorbidities | 1 | Research on integration and coordination between TB and HIV services | 7 | 5 |
|  |  | 2 | Research to evaluate and develop integrated TB services and other services | 12 | 18 |
| 7 | Research to strengthen TB case finding among children | 1 | Research to evaluate and improve capacity on diagnosis and management of pediatric TB | 23 | 9 |
|  |  | 2 | Research to provide and use of children friendly treatment for drug resistant pediatric TB cases | 4 | 0 |
| 8 | Research to explore TB risk factors | 1 | Research to explore risk factors and root causes to increased drug sensitive and resistant TB cases | 17 | 21 |

3

**Research topics proposed by stakeholders related to Strategy 3. Optimising health promotion and TB prevention, TB preventive therapy, and infection control**

|  | **Category** |  | **Sub-categories** | **Number of online survey participants support** | **Number of previous relevant research** |
| --- | --- | --- | --- | --- | --- |
| 1 | Research on TB infection control in healthcare facilities | 1 | Research to evaluated and develop strategies to improve infection control in healthcare facilities | 104 | 0 |
| 2 | Research on the effect of environment and life-styles on TB | 1 | Research on environment management and TB infection control and prevention | 23 | 8 |
|  |  | 2 | Research on the effect of individual behavior and life-styles on TB | 51 | 20 |
| 3 | Research on TB preventive treatment | 1 | Research on TB preventive treatment management | 57 | 2 |
|  |  | 2 | Epidemiological research on latent TB | 21 | 2 |
|  |  | 3 | Research to improve latent TB diagnosis | 3 | 3 |
|  |  | 4 | Development of IEC materials and health promotion for TB prevention | 37 | 0 |
|  |  | 5 | Research on TB preventive treatment among latent TB cases | 1 | 0 |
| 4 | Research to evaluate and intensify contact investigation | 1 | Research to evaluate and intensify contact investigation | 85 | 4 |
|  |  | 2 | Research and innovation on effective strategy of contact investigation | 18 | 1 |

**Research topics proposed by stakeholders related to Strategy 4. Translating research and technology for screening, diagnosis, and treatment**

|  | **Category** |  | **Sub-categories** | **Number of online survey participants support** | **Number of previous relevant research** |
| --- | --- | --- | --- | --- | --- |
| 1 | Research on digital technology of TB program management | 1 | Research on digital TB recording and reporting | 38 | 3 |
|  |  | 2 | Research to simplify forms and TB recording and reporting systems | 20 | 0 |
|  |  | 3 | Research to develop integrated recording and reporting systems between TB and other diseases | 2 | 1 |
|  |  | 4 | Research on digital technology to improve treatment adherence | 9 | 3 |
| 2 | Research to evaluate and develop policy and coordination among ministries and TB research institution | 1 | Research on TB program data utilisation for research and policy development | 18 | 0 |
|  |  | 2 | Research on the expansion of TB research network and mapping of TB research in Indonesia | 1 | 0 |
| 3 | Development of basic research, genetics, clinical research and implementation research | 1 | Development of TB research in Indonesia including operational research, implementation research, genetics, nutrition, and clinical TB research | 4 | 108 |
| 4 | Research on TB vaccine | 1 | Development of new TB vaccine for children and adults | 26 | 1 |
|  |  | 2 | Research to evaluate TB vaccine including distribution and utilisation | 11 | 1 |
| 5 | Research on TB treatment | 1 | Research and development on new TB treatment, dosage evaluation, interactions, side effects, short term treatment, and children friendly treatment | 69 | 35 |
| 6 | Research and development of TB diagnosis | 1 | Research and development of TB diagnosis tools | 16 | 9 |

**Research topics proposed by stakeholders related to Strategy 5. Increasing community and partner engagement in TB elimination**

|  | **Category** |  | **Sub-categories** | **Number of online survey participants support** | **Number of previous relevant research** |
| --- | --- | --- | --- | --- | --- |
| 1 | Research to improve community empowerment through IEC | 1 | Research to evaluate IEC methods and materials | 332 | 3 |
|  |  | 2 | Research to evaluate and improve community engagement in TB control and prevention | 172 | 2 |
| 2 | Research on evaluation and development of partnership and coordination between TB program and other stakeholders | 1 | Research on role of pharmacists, philanthropies, social caritative bodies, and corporate social responsibility to support TB program | 22 | 3 |
|  |  | 2 | Research to evaluation coordination and management of TB program between national, provincial and district governments | 28 | 0 |
| 3 | Research to evaluate and improve feedbacks from community to TB services | 1 | Research to evaluate quality TB services in healthcare facilities | 50 | 2 |
|  |  | 2 | Research to evaluate barriers to access TB services | 9 | 0 |
| 4 | Research to decrease stigma and discrimination to people with TB in community |  | - | 17 | 1 |

**Research topics proposed by stakeholders related to Strategy 6. Improving TB program management through health system strengthening**

|  | **Category** |  | **Sub-categories** | **Number of online survey participants support** | **Number of previous relevant research** |
| --- | --- | --- | --- | --- | --- |
| 1 | Research to evaluate inter-program and inter-sectoral commitment and collaboration among ministries to support TB program |  | - | 35 | 0 |
| 2 | Research to evaluate and develop competencies of TB program human resources | 1 | Research to evaluate availability, workload and need of TB program human resources | 29 | 0 |
|  |  | 2 | Research to evaluate and improve competencies of TB program human resources | 31 | 1 |
|  |  | 3 | Policy analysis related to staff rotation/change, training, remuneration | 29 | 3 |
| 3 | Research to increase quality and sustainability of TB recording and reporting using digital technology |  | - | 26 | 0 |
| 4 | Strengthen financing system for TB service and program | 1 | Research to evaluate and advocate financing for TB program from both national and local governments | 22 | 0 |
|  |  | 2 | Research to evaluate and strengthen TB financing system through national health insurance | 9 | 2 |
|  |  | 3 | Research to evaluate and map potential financing mechanisms for TB program | 8 | 1 |
|  |  | 4 | Research to evaluate TB financing problems | 18 | 1 |
|  |  | 5 | Research to evaluate financing of community activities (including voluntary health cadres) to support TB program | 12 | 7 |
| 5 | Research to improve TB surveillance |  | - | 82 | 0 |
| 6 | Research to evaluate and strengthen TB logistic management | 1 | Research to evaluate and strengthen coordination between Pharmacy Department, District Health Office and Health Facilities in all levels | 13 | 0 |
|  |  | 2 | Research to evaluate planning, mapping and management of TB logistic | 18 | 0 |
